# RegularizedSCA: Regularized simultaneous component analysis of multiblock data in R

**DOI:** 10.3758/s13428-018-1163-z

**Published:** 2018-12-12

**Authors:** Zhengguo Gu, Katrijn Van Deun

**Affiliations:** Department of Methodology and Statistics, TSB, Tilburg University, PO Box 90153, 5000LE Tilburg, The Netherlands

**Keywords:** Common/distinctive components, Group Lasso, Lasso, Linked data analysis, Multiblock analysis, Simultaneous component analysis

## Abstract

This article introduces a package developed for R (R Core Team, 2017) for performing an integrated analysis of multiple data blocks (i.e., linked data) coming from different sources. The methods in this package combine simultaneous component analysis (SCA) with structured selection of variables. The key feature of this package is that it allows to (1) identify joint variation that is shared across all the data sources and specific variation that is associated with one or a few of the data sources and (2) flexibly estimate component matrices with predefined structures. Linked data occur in many disciplines (e.g., biomedical research, bioinformatics, chemometrics, finance, genomics, psychology, and sociology) and especially in multidisciplinary research. Hence, we expect our package to be useful in various fields.

Joint analysis of multiblock data (also referred to as integrated analysis of multiblock data, linked data analysis, or broadly speaking, data fusion; see, Van Mechelen & Smilde, 2010) is getting increasingly popular in recent years. Thanks to modern technology, researchers gather comprehensive data from multiple sources and analyze them jointly. For example, global positioning systems (GPS) data have been combined with self-report travel diary data, and their joint analysis provides a deeper insight into people’s traveling behavior (Mavoa et al., 2011). Social media data such as financial tweets and linked business ontology data have been used to jointly predict the stock market (Sánchez Rada et al., 2014). Other examples can be found in studies on complex interactions between genetic information and environmental conditions (Meloni, 2015), between longitudinal survey data and bio-measures (Buck & McFall, 2011), and between behavioral data (e.g., school census, clinical data) and genetic data (Boyd et al., 2012).

This article introduces an R package for performing joint analysis on large-scale multiblock data from multiple sources. The core algorithms of this package have their roots in traditional simultaneous component analysis (SCA), which has been widely used for performing data integration from multiple sources in biomedical research, bioinformatics, genomics, and psychology (e.g., De Tayrac, Lê, Aubry, Mosser, & Husson, 2009; Gu & Van Deun, 2016; Lock, Hoadley, Marron, & Nobel, 2013; Van Deun, Smilde, van der Werf, Kiers, & Van Mechelen, 2009; Van Deun et al., 2012: Van Deun, Smilde, Thorrez, Kiers, & Van Mechelen, 2013; Wilderjans, Ceulemans, Van Mechelen, & van den Berg, 2011). One may notice that, aside from SCA, other methods, such as canonical correlation analysis (Tenenhaus & Tenenhaus, 2014), may also be used for joint analysis of multiblock data, but we refrain from discussing other methods in this article.

The major advantage of traditional simultaneous component-based data integration methods is that they allow for identifying the same components for all sources, which facilitates joint interpretation across all sources. However, the traditional methods are also limited: First, interpretation of components is based on *all* variables, which makes the results difficult to interpret especially in the case of big data (Van Deun et al., 2011). Second, they are not designed for identifying joint sources of variation that offer shared information across data blocks and identifying unique variation that provides critical information on a few but not all data blocks. An example of joint variation is genes–environment interactions. Researchers are interested in whether specific (susceptible) genes pose a risk in a certain risk-inducing environment, which boils down to linking (a few selected) variables from each of the sources (i.e., genes from the genetic data and environment-relevant variables from the survey data). Here, the linked variables highlight the joint sources of variation in the data. An example of specific variation is personality research in cross-cultural psychology, where specific variation belonging to a particular culture is of substantial interest (Kuppens et al., 2006).

Van Deun et al., (2011) proposed a sparse SCA framework for identifying joint and specific variation in multiblock data. In the sparse SCA framework, the joint and specific variation is manifested in the common and distinctive components of a *component loading matrix*. The interpretation of the component loading matrix is similar to that in principal component analysis (PCA). Based on the sparse SCA framework, Gu and Van Deun (2016) proposed a majorization-minimization (MM) algorithm for identifying common and distinctive components and provided the MATLAB code. Their method performs variable selection by using state-of-the-art penalization methods like the Lasso and thus includes sparse PCA (Shen & Huang, 2008) as a special case. So far, only the MATLAB code implementing the core algorithms is available, which, in essence, makes the method only accessible to those having time, good programming skills, and insight in the method and model selection procedures. Furthermore, the algorithmic approach taken by Gu and Van Deun (2016) has some drawbacks: The MM procedure tends to be slow in searching for the minimum, which may not be desirable in applied research. In addition, MM is an iterative procedure that converges to the optimal solution but, in general, does not reach the optimum *exactly*. For the particular penalized problem considered here and for that studied by Gu and Van Deun (2016), the exact solution to the (conditional) optimization problem can be found by using sub-gradient techniques. With strong penalties, the estimated loadings can be exact zeros instead of approximate zeros, which is what users desire in case of variable selection.

In this article, we introduce the RegularizedSCA package for multiblock data analysis with the capability of identifying joint and specific variation in terms of common and distinctive components, which offers a great interpretational advantage to users. This package incorporates comprehensive algorithms for solving regularized SCA problems and provides user-friendly tools to facilitate model selection and interpretation. In addition, we show that the sparse SCA framework (Van Deun et al., 2011) can be transformed into a sparse Group Lasso regression problem (Yuan & Lin, 2006) resulting in a procedure that not only is more computationally efficient than the MM procedure implemented by Gu and Van Deun (2016) and Van Deun et al., (2011) but can also generate exact zeroes. Furthermore, prior information regarding joint and specific variation in the data can directly be incorporated and analyzed by the algorithms in RegularizedSCA.

An additional bonus of the RegularizedSCA package is that it incorporates several approaches, including DISCO-SCA, the variance accounted for (VAF) method (Schouteden et al., 2014), and the PCA-GCA method (Smilde et al., 2017), to data integration that have been proposed previously but were mainly implemented in MATLAB and not in R. These are all non-sparse approaches and have been included with the purpose of model selection but, if needed, can be used on their own. The existence of multiple approaches is due to the nonunanimously agreed upon concept of joint and specific variation (Smilde et al., 2017). Some researchers focus on the explained variance in each data block (e.g., in psychological research), and therefore joint and specific variation is identified by examining explained variance using, for example, DISCO-SCA together with the VAF method. Others argue that joint and specific variation should be decided based on the correlation between data blocks by using the PCA-GCA method. The RegularizedSCA package includes DISCO-SCA, the VAF method, and the PCA-GCA method to meet researchers’ diverging needs.

In what follows, we first introduce the SCA method, the regularized SCA model, and model selection methods for the regularized SCA model. Next, we illustrate the extensive functionality of RegularizedSCA by using a simple dataset included in the package. Finally, we present an application to joint analysis on three-block survey data on parent–child relationships to help readers understand what kind of research questions in social and behavioral sciences can be answered by using RegularizedSCA.

## Method

### The simultaneous component analysis (SCA) model

SCA can be regarded as an extension of principal component analysis (PCA). Suppose *K* data blocks are to be integrated, and let **X**_*k*_, a *I*_*k*_ × *J*_*k*_ matrix, denote the *k* th (*k* = 1,2,...,*K*) data block containing scores of *I*_*k*_ subjects, objects, or experimental conditions on *J*_*k*_ variables. Based on PCA, **X**_*k*_ can be decomposed as follows

1 $$ \mathbf{X}_{k}=\mathbf{T}_{k}\mathbf{P}^{T}_{k}+\mathbf{E}_{k}, $$

with *R* components. The component score matrix **T**_*k*_ is of size *I*_*k*_ × *R*; the matrix of component loadings **P**_*k*_ is of size *J*_*k*_ × *R*. **E**_*k*_ denotes the residuals. To identify the solution, extra constraints, such as $\mathbf {T}^{T}_{k}\mathbf {T}_{k}=\mathbf {I}$ and a principal axis orientation, are assumed (Van Deun et al., 2009).

Unlike PCA, which focuses on one data block, SCA analyzes multiple data blocks altogether. Since data blocks are integrated with respect to the same rows, we have *I*_1_ = ⋯ = *I*_*k*_ = ⋯ = *I*_*K*_ = *I*. SCA requires that the component scores should be the same across all data blocks, and thus for each data block

2 $$ \mathbf{X}_{k}=\mathbf{T}\mathbf{P}^{T}_{k}+\mathbf{E}_{k}, $$

with **T**^*T*^**T** = **I** and **T**_1_ = ⋯ = **T**_*k*_ = ⋯ = **T**_*K*_ = **T**. Estimates of the model (2) can be obtained by solving the following least squares minimization problem

3 $$ \underset{\mathbf{T}, \mathbf{P}_{k}}{\min}\sum\limits_{k}\|\mathbf{X}_{k}-\mathbf{T}\mathbf{P}^{T}_{k}\|^{2}_{2} $$

under the constraints. Optimal **T** and **P**_*k*_ for Eq. 3 can be obtained from the singular value decomposition (SVD) of the concatenated data [**X**_1_⋯**X**_*K*_] (see, e.g., Van Deun et al., 2009).

#### Common and distinctive components

The SCA model cannot be used to identify joint and specific variation in data, which is well known in psychometrics (Smilde et al., 2017) for data integration. Schouteden et al., (2013) and Schouteden et al., (2014) proposed the DISCO-SCA method, which involves rotating the SCA solution to common and distinctive components by introducing a target matrix that defines distinctive components by loadings that are zero everywhere except for the variables of the block(s) that they are supposed to underlie; common components are left unspecified in the target. In general, the rotated loadings will not result in zero or close-to-zero loadings for the distinctive components and loadings that are (much) higher in absolute value than zeros for the common component. To check if the rotated loadings are indeed distinctive, the proportion of VAF by the component in each of the blocks is computed: if this proportion is considerably higher in the block(s) underlying the component than in the other blocks, the component is called distinctive according to the DISCO-SCA method; if the proportion is approximately the same in all blocks, the component is called common.

**Fig. 1:**
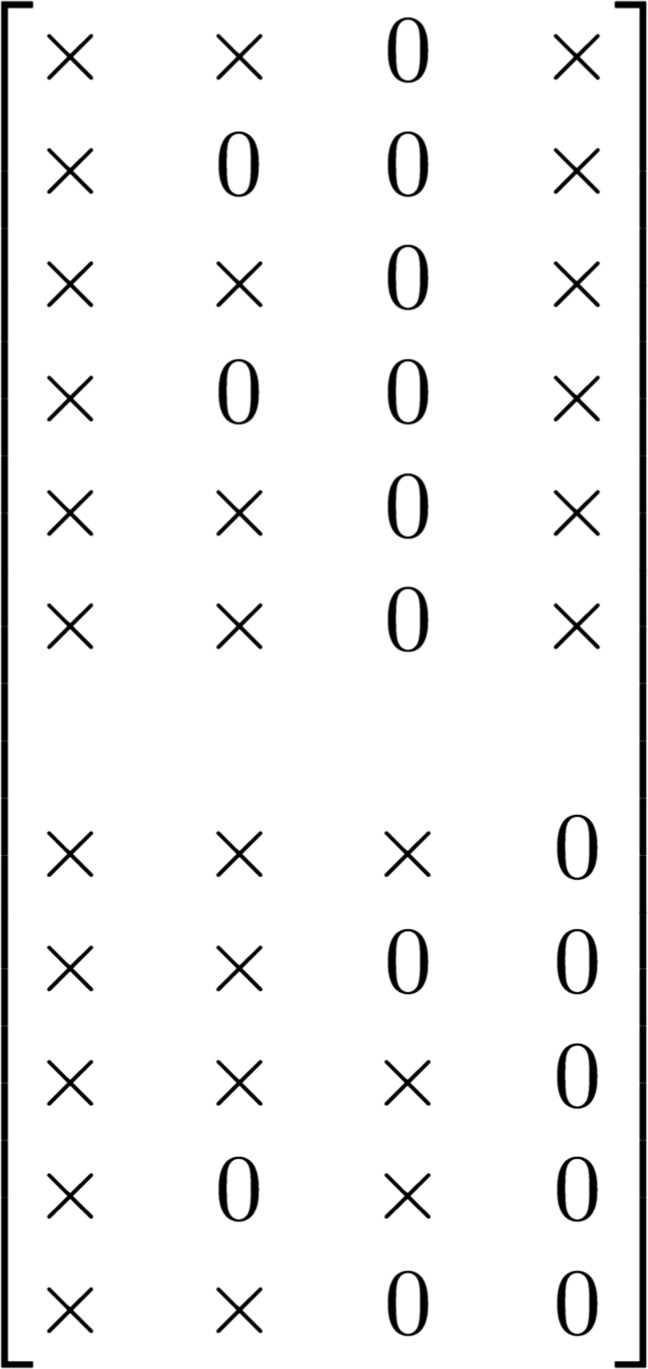
An example of common/distinctive components in a concatenated component loading matrix. The columns represent components, and the rows represent variables. The first six rows contain loadings from the first data block, and the remaining five rows contain loadings from the second data block. “×” indicates a non-zero loading, and “0” denotes a zero loading

In this article, we give a formal definition of common and distinctive components as follows. For the *r* th component (*r* = 1,2,...,*R*), its component loading vector corresponding to the *k* th data block is denoted by $\mathbf {p}^{k}_{r}$. The *r* th component is referred to as a common component across all data blocks, if $\mathbf {p}^{k}_{r} \neq \mathbf {0}$ (i.e., *at least* one loading in the *r* th component belonging to the *k* th block is not zero) for all *k*(*k* = 1,...,*K*). Figure 1 presents a schematic view of a concatenated component loading matrix from two data blocks with four components (i.e., columns), where “×” denotes a non-zero loading, and “0” denotes a zero loading. In Fig. 1, the first column is such a common component, which we refer to as a “non-sparse” common component. Common components reflect the joint variation across all data blocks. The *r* th component is referred to as a distinctive component, if for some *k*, $\mathbf {p}^{k}_{r} = \mathbf {0}$ (i.e., *all* loadings in the *r* th component belonging to the *k* th block are zero). In Fig. 1, the fourth column is a “non-sparse” distinctive component. Distinctive components reflect specific variation presented in some, but not all, data blocks. The second and third columns in Fig. 1 are referred to as “sparse” common and distinctive components, discussed shortly in the next subsection.

Defining common and distinctive components with respect to **P**_*k*_(*k* = 1,...,*K*), as we do here, gives a very clear meaning to the components. Zero loadings suggest that the corresponding variables are not associated to the component, whereas variables with non-zero loadings are associated to the component. These components represent structural sources of variation, and components with linked variables across data blocks represent joint variation while components with non-zero loadings for variables of only one or a few blocks represent specific sources of variation. Unfortunately, the SCA model does not yield such structures of zero loadings (Van Deun et al., 2011), and therefore regularization is introduced to the SCA model.

### The regularized SCA models

Throughout this section, we assume that the total number of components for all data blocks, *R*, is known, and we will discuss how to obtain *R* in the Model Selection section. We distinguish two situations. Situation (1): We do not know the specific component structure; that is, for each component, we do not know whether zero loadings are fixed for a single block or for multiple but not all blocks (i.e., a distinctive component) or whether all blocks contain non-zero loadings (i.e., a common component). Situation (2): We know the specific component structure (based on, for example, existing literature); that is, we know the position of zero loadings defining the distinctive structure but we need to estimate the remaining undefined (non-zero) loadings.

#### Regularized SCA model with unknown component structure

Building upon sparse principal component analysis (Zou et al., 2006) and simultaneous component analysis, the regularized SCA (also called sparse SCA) model was proposed by Van Deun et al., (2011) and extended by Gu and Van Deun (2016) to component-specific penalties (see Eq. 4 below). The latter extension was needed to allow for solutions with a mix of common and distinctive components. The regularized SCA model is capable of identifying common components (i.e., joint variation) in the component loading matrix across all data blocks and distinctive components (i.e., specific variation) that belong to one or a few blocks. The regularized SCA model can identify non-sparse common components (e.g., the first column in Fig. 1), sparse common components (e.g., the second column), sparse distinctive components (e.g., the third column), and non-sparse distinctive components (e.g., the fourth column).

The regularized SCA model minimizes the following objective function

4 $$ \underset{\mathbf{T}, \mathbf{P}_{k}}{\min}\sum\limits_{k}\|\mathbf{X}_{k}-\mathbf{T}\mathbf{P}^{T}_{k}\|^{2}_{2} + \lambda_{L}\sum\limits_{k}\|\mathbf{P}_{k}\|_{1} + \lambda_{G}\sum\limits_{k}\sqrt{J_{k}}\|\mathbf{P}_{k}\|_{2} $$

subject to

$$\mathbf{T}^{T}\mathbf{T}=\mathbf{I}; \lambda_{L}, \lambda_{G}\geq0, $$

where ${\sum }_{k}\|\mathbf {P}_{k}\|_{1} = {\sum }_{k}{\sum }_{j_{k},r}|p_{j_{k} r}|$ is the Lasso penalty, ${\sum }_{k}\sqrt {J_{k}}\|\mathbf {P}_{k}\|_{2} = {\sum }_{k}\sqrt {J_{k}{\sum }_{j_{k},r}(p^{2}_{j_{k}r})}$ is the Group Lasso penalty, and $p_{j_{k}r}$ denotes the element on the *j* th row and *r* th column in matrix **P**_*k*_. All the variables in **X**_*k*_ are mean-centered and scaled to norm one, which is a commonly used pre-processing step (see, e.g., Gu & Van Deun, 2016; Van Deun et al., 2011). Note that the penalties ${\sum }_{k}\|\mathbf {P}_{k}\|_{1}$ and ${\sum }_{k}\sqrt {J_{k}}\|\mathbf {P}_{k}\|_{2}$ result in shrinkage of coefficients associated to loadings and a block of loadings to zero, respectively. The amount of shrinkage and zero loadings is tuned by the tuning parameter *λ*_*L*_ for the Lasso and by the tuning parameter *λ*_*G*_ for the Group Lasso. As a side note, by introducing penalties, the regularized SCA model may suffer from some loss in fit of the solution to the data, which is not the case for the DISCO-SCA method mentioned previously. The minimization problem (4) can be rewritten in the following vectorized form

5 $$\begin{array}{@{}rcl@{}} \!\!\!\!&&\underset{\mathbf{T}, \mathbf{P}_{k}}{\min}\!\sum\limits_{k}\|\text{vec}(\mathbf{X}_{k}) - (\mathbf{I}\!\otimes\mathbf{T})\text{vec}(\mathbf{P}^{T}_{k})\|^{2}_{2} + \lambda_{L}\!\sum\limits_{k}\|\text{vec}(\mathbf{P}_{k})\|_{1}\\ \!\!\!\!&&+ \lambda_{G}\sum\limits_{k}\sqrt{J_{k}}\|\text{vec}(\mathbf{P}_{k})\|_{2} \end{array} $$

subject to

$$\mathbf{T}^{T}\mathbf{T}=\mathbf{I}; \lambda_{L}, \lambda_{G}\geq0. $$

To solve the minimization problem (5), **T** and **P**_*k*_ are estimated iteratively until convergence, given *R* components, *λ*_*L*_, and *λ*_*G*_ (i.e., pre-specified *R*, *λ*_*L*_, and *λ*_*G*_). We discuss how to identify *R*, *λ*_*L*_, and *λ*_*G*_ in the Model Selection section below. **T** is estimated by **T** = **VU**^*T*^, where **U**Σ**V**^*T*^ is the SVD of $\mathbf {P}^{T}_{C}\mathbf {X}^{T}_{C}$. Here, **P**_*C*_ is the concatenated component loading matrix consisting of *K* blocks of component loadings **P**_*k*_, and **X**_*C*_ is the concatenated data consisting of *K* blocks. To estimate **P**_*k*_, Gu and Van Deun (2016) proposed to use the MM procedure; that is, they replaced (5) with a surrogate function

$$\begin{array}{@{}rcl@{}} \!\!\!\!&& \underset{\mathbf{T}, \mathbf{P}_{k}}{\min}\!\sum\limits_{k}\!\|\text{vec}(\mathbf{X}_{k}) - (\mathbf{I}\otimes\mathbf{T})\text{vec}(\mathbf{P}^{T}_{k})\|^{2}_{2} + \lambda_{L}\sum\limits_{k}\|\text{vec}(\mathbf{P}_{k})\|_{1} \\ \!\!\!\!&& + \sum\limits_{k} \left\{ \frac{\lambda_{G}}{2}\text{vec}(\mathbf{P}^{T}_{k})^{T}\sqrt{J_{k}}\textbf{D}\left\{m^{G}_{kr}\right\}\text{vec}(\mathbf{P}^{T}_{k})\right\}\\ \!\!\!\!&& + \frac{\lambda_{G}}{2}\sum\limits_{k,r}\left( m^{G}_{kr}\right)^{-1}, \end{array} $$

where **D**{*x*} denotes a diagonal matrix with element *x* on its diagonal, and $m^{G}_{kr}=\left ({\sum }_{j_{k}}\|p^{(o)}_{j_{k} r}\|^{2}\right )^{-1/2}$, which is a scalar depending on the current estimate of the component loadings in **P**_*k*_ (i.e., $p^{(o)}_{j_{k} r}$). Note that the particular MM iterations here are such that no exact zeros can result from the Group Lasso penalty, although, with sufficiently high *λ*_*G*_, these are the solutions to the optimization problem. Gu and Van Deun (2016) used rounding (with some arbitrary cut-off) at termination of the MM procedure, despite that, strictly speaking, exact zeros were needed to obtain distinctive components.

We now show that Eq. 5 does not require the MM procedure. **P**_*k*_ can be solved by noticing that the minimization problem (5) is in fact a special case of sparse Group Lasso, and its solution (i.e., estimated component loading matrix $\hat {\mathbf {P}}_{k}$) is directly derived from Yuan and Lin (2006) and Friedman et al., (2010). To see this, notice that the objective function (4) with respect to **P**_*k*_ is

6 $$\begin{array}{@{}rcl@{}} \!\!&& \underset{\mathbf{P}_{k}}{\min}\sum\limits_{l\neq k}\|\mathbf{X}_{l} - \mathbf{T}\mathbf{P}^{T}_{l}\|^{2}_{2} + \|\mathbf{X}_{k} - \mathbf{T}\mathbf{P}^{T}_{k}\|^{2}_{2} + \lambda_{L}\sum\limits_{l \neq k}\|\mathbf{P}_{l}\|_{1} \\ \!\!&& + \lambda_{L}\|\mathbf{P}_{k}\|_{1} + \sum\limits_{l \neq k}\lambda_{G}\sqrt{J_{l}}\|\mathbf{P}_{l}\|_{2} + \lambda_{G}\sqrt{J_{k}}\|\mathbf{P}_{k}\|_{2} \end{array} $$

7 $$\begin{array}{@{}rcl@{}} \!\!&\Rightarrow & \underset{\mathbf{P}_{k}}{\min} \|\mathbf{X}_{k} - \mathbf{T}\mathbf{P}^{T}_{k}\|^{2}_{2} + \lambda_{L}\|\mathbf{P}_{k}\|_{1} + \lambda_{G}\sqrt{J_{k}}\|\mathbf{P}_{k}\|_{2} \end{array} $$

8 $$\begin{array}{@{}rcl@{}} \!\!&= & \underset{\mathbf{P}_{k}}{\min} \|\text{vec}(\mathbf{X}_{k}) - (\mathbf{I}\!\otimes\! \mathbf{T})\text{vec}(\mathbf{P}^{T}_{k})\|^{2}_{2} + \lambda_{L}\|\mathbf{P}_{k}\|_{1} + \lambda_{G}\sqrt{J_{k}}\|\mathbf{P}_{k}\|_{2}. \end{array} $$

Note that Eq. 8 is in the form of a regression problem, where vec(**X**_*k*_) plays the role of the outcome, **I** ⊗**T** of the predictors, and $\text {vec}(\mathbf {P}^{T}_{k})$ of the regression weights. Because (**I** ⊗**T**)^*T*^(**I** ⊗**T**) = **I** (i.e., the predictors are independent), Eq. 8 is a sparse group Lasso problem whose standard solution is given by Yuan and Lin (2006). It can be shown that the solution to Eq. 8 is

9 $$\begin{array}{@{}rcl@{}} \text{vec}(\hat{\mathbf{P}}^{T}_{k}) &=& \left[\frac{1}{2}-\frac{\lambda_{G}\sqrt{J_{k}}}{2\|\mathcal{S}\left( 2(\mathbf{I}\otimes\mathbf{T})^{T}\text{vec}(\mathbf{X}_{k}), \lambda_{L}\right)\|_{2}}\right]_{+}\\ &&\times \mathcal{S}\left( 2(\mathbf{I}\otimes\mathbf{T})^{T}\text{vec}(\mathbf{X}_{k}), \lambda_{L}\right). \end{array} $$

$\mathcal {S}(\cdot )$ is the soft-thresholding operator. [*x*]_+_ = *x*, if *x* > 0; [*x*]_+_ = 0, if *x* ≤ 0. Equation 9 is a closed form solution (unlike the solution based on the MM procedure) for one group of coefficients. To solve for all groups, the algorithm iterates over each group. Notice that in Eq. 8, the Group Lasso penalty is imposed on the entire component matrix **P**_*k*_, which we refer to as the block-wise method. Equation 9 is informative on how the sparseness is achieved. The first half of Equ. 9, $\left [1/2-(\lambda _{G}\sqrt {J_{k}})/\right .$$\left .\left (2\|\mathcal {S}\left (2(\mathbf {I}\otimes \mathbf {T})^{T}\text {vec}(\mathbf {X}_{k}), \lambda _{L}\right )\|_{2}\right )\right ]_{+}$, dictates whether an entire block of component loadings should be replaced with zeros, and if $\left [1/2-(\lambda _{G}\sqrt {J_{k}})/\right .$$\left .\left (2\|\mathcal {S}\left (2(\mathbf {I}\otimes \mathbf {T})^{T}\text {vec}(\mathbf {X}_{k}), \lambda _{L}\right )\|_{2}\right )\right ]_{+} > 0$, then the second half, $\mathcal {S}\left (2(\mathbf {I}\otimes \mathbf {T})^{T}\text {vec}(\mathbf {X}_{k}), \lambda _{L}\right )$, works as a shrinkage operator within the entire block of component loadings and shrinks some but not all loadings to zeros.

Alternatively, the Group Lasso penalty can be imposed on the *r* th component (*r* = 1,2,...,*R*), denoted by $\mathbf {p}^{k}_{r}$, of **P**_*k*_, resulting in the component-wise method. In this case, starting from Eq. 7, we solve the following minimization problem with respect to $\mathbf {p}^{k}_{r}$

10 $$\begin{array}{@{}rcl@{}} &&\underset{\mathbf{p}^{k}_{r}}{\min}\|\mathbf{X}_{k} - \mathbf{T}\mathbf{P}^{T}_{k}\|^{2}_{2} + \lambda_{L}\sum\limits^{R}_{r = 1}\|\mathbf{p}^{k}_{r}\|_{1} \!\!+ \lambda_{G}\sqrt{J_{k}}\sum\limits^{R}_{r = 1}\|\mathbf{p}^{k}_{r}\|_{2} \end{array} $$

11 $$\begin{array}{@{}rcl@{}} &= &\underset{\mathbf{p}^{k}_{r}}{\min}\|\mathbf{X}^{T}_{k} - \sum\limits_{r = 1}^{R}\mathbf{p}^{k}_{r}\mathbf{t}^{T}_{r}\|^{2}_{2}\!+ \!\lambda_{L}\sum\limits^{R}_{r = 1}\|\mathbf{p}^{k}_{r}\|_{1} + \lambda_{G}\sqrt{J_{k}}\sum\limits^{R}_{r = 1}\|\mathbf{p}^{k}_{r}\|_{2}, \end{array} $$

where **t**_*r*_ is the *r* th column in **T**. Let

$$ \mathbf{R}_{k} := \mathbf{X}^{T}_{k} - \sum\limits_{s \neq r}^{R}\mathbf{p}^{k}_{s}\mathbf{t}^{T}_{s}, $$

and then Eq. 11 becomes

12 $$\begin{array}{@{}rcl@{}} \!\!&& \underset{\mathbf{p}^{k}_{r}}{\min}\|\mathbf{R}_{k} - \mathbf{p}^{k}_{r}\mathbf{t}^{T}_{r}\|^{2}_{2}+ \lambda_{L}\sum\limits^{R}_{r = 1}\|\mathbf{p}^{k}_{r}\|_{1} + \lambda_{G}\sqrt{J_{k}}\sum\limits^{R}_{r = 1}\|\mathbf{p}^{k}_{r}\|_{2} \end{array} $$

13 $$\begin{array}{@{}rcl@{}} \!\!&\Rightarrow & \underset{\mathbf{p}^{k}_{r}}{\min}\|\mathbf{R}_{k} - \mathbf{p}^{k}_{r}\mathbf{t}^{T}_{r}\|^{2}_{2}+ \lambda_{L}\|\mathbf{p}^{k}_{r}\|_{1} + \lambda_{G}\sqrt{J_{k}}\|\mathbf{p}^{k}_{r}\|_{2} \end{array} $$

14 $$\begin{array}{@{}rcl@{}} \!\!&= & \underset{\mathbf{p}^{k}_{r}}{\min}\|\text{vec}(\mathbf{R}_{k}) - (\mathbf{t}_{r} \otimes \mathbf{I})\mathbf{p}^{k}_{r}\|^{2}_{2} + \lambda_{L}\|\mathbf{p}^{k}_{r}\|_{1} + \lambda_{G}\sqrt{J_{k}}\|\mathbf{p}^{k}_{r}\|_{2}. \end{array} $$

Because (**t**_*r*_ ⊗**I**)^*T*^(**t**_*r*_ ⊗**I**) = **I**, it can be proven that

15 $$\begin{array}{@{}rcl@{}} \hat{\mathbf{p}}^{k}_{r} &=& \left[\frac{1}{2} - \frac{\lambda_{G}\sqrt{J_{k}}}{2\|\mathcal{S}\left( 2(\mathbf{t}_{r}\otimes\mathbf{I})^{T}\text{vec}(\mathbf{R}_{k}), \lambda_{L}\right)\|_{2}} \right]_{+}\\ &&\times \mathcal{S}\left( 2(\mathbf{t}_{r}\otimes\mathbf{I})^{T}\text{vec}(\mathbf{R}_{k}), \lambda_{L}\right), \end{array} $$

see Yuan and Lin (2006). The first half of Eq. 15, $\left [1/2 - (\lambda _{G}\sqrt {J_{k}})/(2\|\mathcal {S}\left (2(\mathbf {t}_{r}\otimes \mathbf {I})^{T}\text {vec}(\mathbf {R}_{k}), \lambda _{L}\right )\|_{2}) \right ]_{+}$, decides whether an entire component in a block should be replaced with zeros, and if $\left [1/2 - (\lambda _{G}\sqrt {J_{k}})/\right .$$\left .(2\|\mathcal {S}\left (2(\mathbf {t}_{r}\otimes \mathbf {I})^{T}\text {vec}(\mathbf {R}_{k}), \lambda _{L}\right )\|_{2}) \right ]_{+} >0$, then the second half $\mathcal {S}\left (2(\mathbf {t}_{r}\otimes \mathbf {I})^{T}\text {vec}(\mathbf {R}_{k}), \lambda _{L}\right )$ searches through the component and shrinks some (but not all) loadings to zeros. We present the algorithm for solving the regularized SCA model with unknown component structure in Appendix (see Algorithm 1).

Our experience is that the component-wise method is more useful in practice, because by imposing Group Lasso penalties on each component of each block, the common and distinctive components are directly identified. The block-wise method is useful when users are not sure whether certain data blocks provide any information at all—if not, the entire data blocks can be dropped from analysis. In the remainder of this article, we focus on the use of the component-wise method, but the block-wise method is also mentioned when necessary.

Because the sparse group lasso regression satisfies the Karush–Kuhn–Tucker (KKT) conditions (Yuan & Lin, 2006), the convergence is guaranteed for each iteration where **P**_*C*_ is updated. **P**_*C*_ and **T** are updated iteratively, and this procedure guarantees that the loss is non-increasing, but local minima rather than the global minimum might be attained. Thus, Algorithm 1 (see the Appendix) is combined with a multi-start procedure; that is, the algorithm is repeated multiple times with different starting values of **P**_*C*_. It should be noted that the running time of the algorithm increases because of the multi-start procedure. In the RegularizedSCA package, users can freely decide the number of random starts for the multi-start procedure. In addition, due to the regularization penalties, the non-zero component loadings are closer to zero than if there would be no penalties. If desired, one may also undo the shrinkage by re-estimating the non-zero loadings by means of OLS (Gu & Van Deun, 2016).

#### Regularized SCA model with known component structure

Sometimes, a researcher may know the general component structure *a priori*; that is, she/he knows for each component whether it is common or distinctive for one or a few particular blocks. In such circumstances, what interests a researcher often is that, whether it is possible to achieve a higher level of sparseness. For example, suppose previous research suggests that there are two components, one of which is a non-sparse common component like the first column in Fig. 1 and the other of which is a non-sparse distinctive component like the fourth column in the same figure. Can we further introduce some sparseness to the two non-sparse components by turning them into sparse common and distinctive components like the second and third columns in Fig. 1? In this case, one may fix the zero loadings that are known *a priori* and let the algorithm estimate the remaining loadings freely. To fix the zero loadings, the RegularizedSCA package requires users to enter a so-called target matrix, which contains the information of the specific component structure known to users. How to specify the target matrix is explained in “The RegularizedSCA package” Section below. Also, because the specific component structure is known, the Group Lasso penalty, which originally is included to identify the component structure (i.e., by suppressing component loadings of a component or a block to zeros), is not needed. Thus, the minimization problem (5) simplifies to a Lasso regression problem, and the Group Lasso penalty is removed (i.e., *λ*_*G*_ = 0; Gu & Van Deun, 2016). In fact, we can consider this Lasso regression problem as a special, component-wise case of the regularized SCA model with unknown component structure (see Eq. 15): The difference is that, when the component structure is known, the first half of Eq. 15, $\left [1/2 - (\lambda _{G}\sqrt {J_{k}})/(2\|\mathcal {S}\left (2(\mathbf {t}_{r}\otimes \mathbf {I})^{T}\text {vec}(\mathbf {R}_{k}), \lambda _{L}\right )\|_{2}) \right ]_{+} \equiv 1/2$ (because *λ*_*G*_ = 0), and the remaining half of Eq. 15, $\mathcal {S}\left (2(\mathbf {t}_{r}\otimes \mathbf {I})^{T}\text {vec}(\mathbf {R}_{k}), \lambda _{L}\right )$, is the standard solution to a Lasso regression problem. The Lasso regression satisfies the KKT conditions (Hastie, Tibshirani, & Wainwright, 2015, p. 9), and therefore the convergence is guaranteed for each iteration where **P**_*C*_ is updated. Because the loss function is biconvex, convergence is thus to a local minimum and the algorithm also requires a multi-start procedure. In addition, one may undo the shrinkage of the non-zero loadings by means of OLS (Gu & Van Deun, 2016). We present the algorithm for solving the regularized SCA model with known component structure in the Appendix (see, Algorithm 2).

### Model selection

**Fig. 2:**
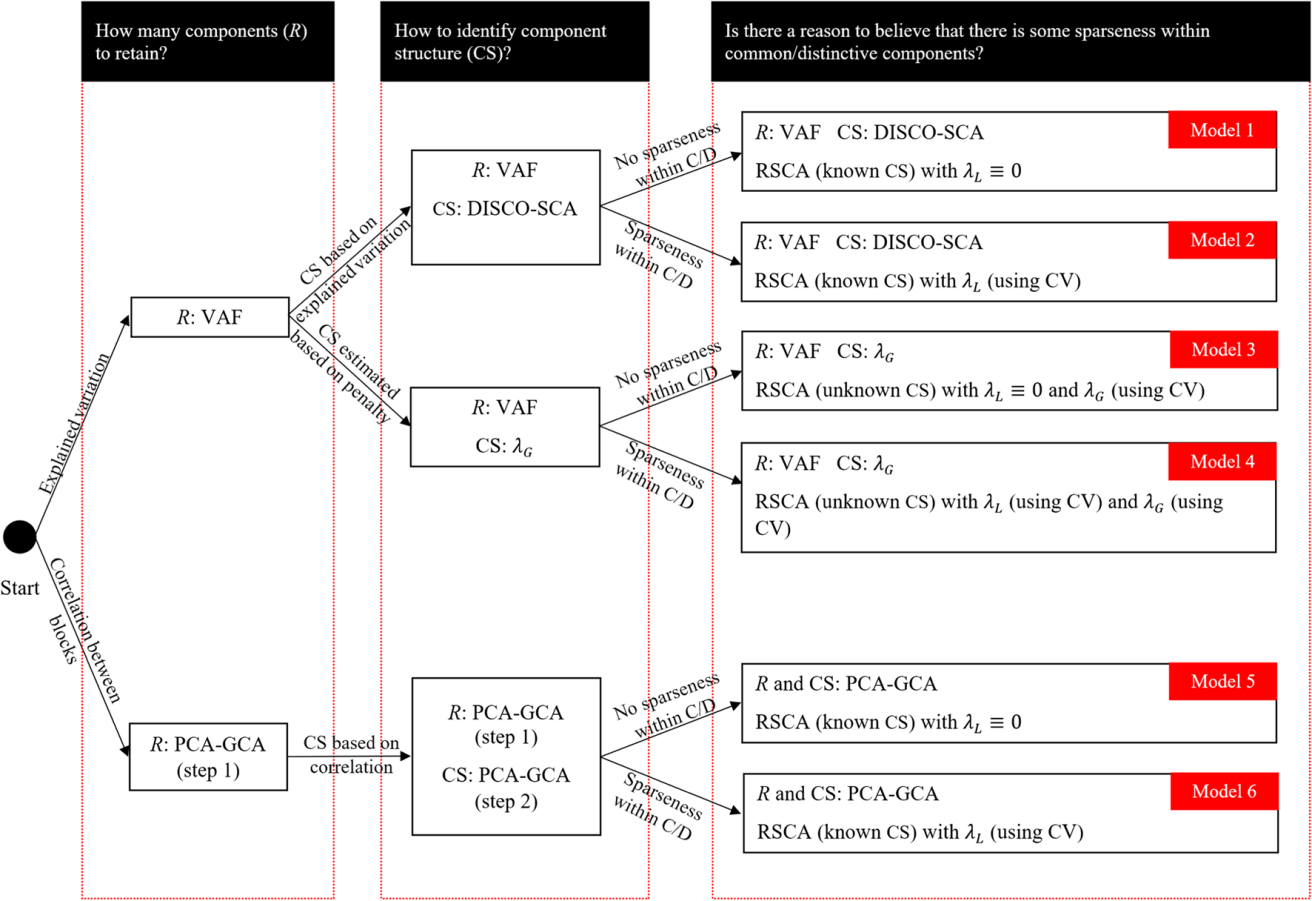
A flow chart for model selection. Note that CS stands for component structure, and C/D stands for common and distinctive components

The regularized SCA model (5) with unknown component structure is formulated with a fixed number of components (i.e., *R*) and fixed values for the tuning parameters for the Lasso and Group Lasso (i.e., *λ*_*L*_ and *λ*_*G*_). The regularized SCA model with known component structure (i.e., Eq. 5 with *λ*_*G*_ = 0) is formulated with a fixed number of *R* and a fixed value for *λ*_*L*_. To help users choose the most suitable model, we present a flow chart (Fig. 2). Users are advised to ask themselves three questions, which are (1) “How many components (*R*) for all blocks to retain?”, (2) “How to identify the component structure, given *R*?”, and (3) “Is there a reason (e.g., according to previous research) to believe that there is some sparseness within common/distinctive components?” Depending on the answers to the questions, one chooses a model in Fig. 2.

#### Deciding the number of components *R* for all data blocks

The RegularizedSCA package provides the VAF method and the PCA-GCA method for identifying the number of components. The VAF method computes the proportion of VAF for each simultaneous component in each data block (Schouteden et al., 2013; Schouteden et al., 2014). One may perform simultaneous component analysis without Lasso and Group Lasso penalties given an arbitrarily large number of components *R*^∗^ >> *R* and compute the VAF for each component in each block. According to Schouteden et al. (2013, 2014), one may choose a proper value for *R* such that the VAF for the first *R* components is clearly higher than for the remaining (*R*^∗^− *R*) components in *any* block.

The PCA-GCA method (for details, see, Smilde et al., 2017) works in two steps. The first step concerns identifying the number of components for *each* data block. In this step, PCA is performed separately on each block, and the appropriate number of components for each block is identified (via, for example, a scree plot). However, deciding *R* for *all* data blocks by means of the PCA-GCA method requires a second step (discussed below); that is, *R* is obtained once the component structure is identified.

#### Identifying the component structure

Several tools are available for this purpose. The first tool is the DISCO-SCA method (Schouteden et al., 2013, 2014), provided that the VAF method has been used for deciding *R*. In a nutshell, DISCO-SCA is performed in such a way that **P**_*C*_ is rotated towards a sequence of so-called target matrices, each target matrix corresponding to a possible combination of common and distinctive components. Since **P**_*C*_ is rotated, **T** will be rotated accordingly. The best combination of common and distinctive components is identified by certain rules based on the sum of squared scores of components (see, Schouteden et al., 2013, for details). We emphasize that, in RegularizedSCA, the DISCO-SCA method is not used to estimate common and distinctive components but is used to identify the component structure. As a side note, readers may wonder why not first let DISCO-SCA identify the component structure, then the sum of the number of common/distinctive components is *R*. However, this is impossible, because the procedure (Schouteden et al., 2013, 2014) was proposed as a stepwise method where first *R* has to be determined and next the common/distinctive structure is determined by checking all possible configurations of common and distinctive components for *R*.

The second tool is the PCA-GCA method, provided that the same method has been used for deciding the number of components for each data block. To identify the component structure, generalized canonical correlation analysis (GCA) is performed on the component scores of every two data blocks to identify the number of common components. A common component is identified if the correlation is above a threshold. For example, Smilde et al. (2017, p. 15) used .7 as the correlation threshold for the medical biology data. Once the number of common components is identified, the rest are the distinctive components, and thus the component structure is identified.

The third tool requires the Group Lasso penalty by means of the component-wise method, provided that *R* has been decided by the VAF method. In this case, we let the algorithm identify a suitable component structure for us by identifying *λ*_*G*_ via cross-validation.

#### Sparseness *within* common/distinctive components

Once a component structure is identified, one may decide whether there is sparseness within the common/distinctive components; that is, whether the components should look like the first and fourth columns in Fig. 1 or look like the second and third columns in the figure. The sparseness within common/distinctive components is achieved by using a Lasso penalty with *λ*_*L*_≠ 0, and *λ*_*L*_ is identified by means of a cross-validation procedure. By giving answers to the three questions, users choose one of the six models in Fig. 2. Take models 1 and 2 in the figure for example. When *R* is decided by means of the VAF method, and the component structure is identified by means of DISCO-SCA, the recommended model is the regularized SCA model with known structure with *λ*_*L*_ ≡ 0, if it is believed that there is no sparseness within common/distinctive components (i.e., Model 1). Alternatively, one may first use Model 2 and check whether the cross-validation procedure recommends a very small *λ*_*L*_. A very small *λ*_*L*_ may suggest that there is little support for a sparse model, and therefore one may use Model 1 instead. If one intends to achieve some sparseness within common/distinctive components, then Model 2 is preferred.

Note that models 2, 3, 4, and 6 incorporate a *K*-fold cross-validation procedure for identifying the optimal *λ*_*L*_ and/or *λ*_*G*_. When both *λ*_*L*_ and *λ*_*G*_ are used (i.e., Model 4), the algorithm searches through a grid of *λ*_*L*_ and *λ*_*G*_ values, and for each pair of *λ*_*L*_ and *λ*_*G*_, *K*-fold cross-validation is performed. Take 10-fold cross-validation for example, 10% of the cells from the data are replaced with missing values, which are then replaced with the mean across subjects that do not contain missing values. The optimal combination of *λ*_*L*_ and *λ*_*G*_ is identified as follows. First, the algorithm computes mean squared prediction errors (James, Witten, Hastie, & Tibshirani, 2013, p. 181) given each combination of *λ*_*L*_ and *λ*_*G*_. Let MSPE(*λ*_*L*_,*λ*_*G*_) denote the mean squared prediction error given *λ*_*L*_ and *λ*_*G*_. Let $(\lambda ^{*}_{L}, \lambda ^{*}_{G})$ denote the combination that generates the lowest mean squared prediction error. Second, the algorithm computes the sample standard deviation in the *K* estimates of the prediction error associated to $(\lambda ^{*}_{L}, \lambda ^{*}_{G})$ (i.e., the standard error in the estimates of the prediction error for $(\lambda ^{*}_{L}, \lambda ^{*}_{G})$), denoted by $\text {SE}(\lambda ^{*}_{L}, \lambda ^{*}_{G})$. Finally, the optimal combination of *λ*_*L*_ and *λ*_*G*_, denoted by $({\lambda ^{o}_{L}},{\lambda ^{o}_{G}})$, is the one for which the mean squared prediction error $\text {MSPE}({\lambda ^{o}_{L}}, {\lambda ^{o}_{G}})$ is closest to (but not higher than) $\text {MSPE}(\lambda ^{*}_{L}, \lambda ^{*}_{G}) +\text {SE}(\lambda ^{*}_{L}, \lambda ^{*}_{G})$. This method is referred to as the “one standard error rule” recommended by Hastie et al. (2015, p. 13). When only *λ*_*L*_ or *λ*_*G*_ is used (i.e., models 2, 3, 6), the algorithm searches through a sequence of *λ*_*L*_ or *λ*_*G*_, and the optimal *λ*_*L*_ or *λ*_*G*_ is also obtained based on the “one standard error rule”. For example, when only *λ*_*L*_ is used, the algorithm first computes the mean squared prediction errors and looks for the lowest mean squared prediction error, denoted by $\text {MSPE}(\lambda ^{*}_{L})$. Then, the algorithm computes the standard error associated to $\lambda ^{*}_{L}$, denoted by $\text {SE}(\lambda ^{*}_{L})$. The optimal ${\lambda ^{o}_{L}}$ is the one for which the mean squared prediction error $\text {MSPE}({\lambda ^{o}_{L}})$ is closest to (but not higher than) $\text {MSPE}(\lambda ^{*}_{L})+\text {SE}(\lambda ^{*}_{L})$. For detailed explanations about cross-validation and its application to sparse models, we recommend James et al., (2013) and Witten et al., (2009), and in the context of component models we recommend Bro et al., (2008).

The package includes the VAF method, DISCO-SCA, and the PCA-GCA method, because they represent two different view points in multi-block data research (Smilde et al., 2017). The VAF method and DISCO-SCA focus on explained variation in each data block, whereas the PCA-GCA method emphasizes the correlation between data blocks. Since they follow different approaches, we do not expect them to always generate the same *R* and identify the same component structure. We advise readers to choose one of the two approaches, depending on their research fields and/or existing research. It is possible to establish a cross-validation procedure for deciding *R*, *λ*_*L*_, and *λ*_*G*_ altogether in one step (and therefore the VAF method, the DISCO-SCA method, and the PCA-GCA method are no longer needed). However, such a comprehensive cross-validation procedure is computationally expensive and still too immature to be included in RegularizedSCA, because such a procedure requires an algorithm to search through a three-dimensional grid using a multi-start procedure. Studying the usefulness of such comprehensive procedures is much needed and deserves full attention in a separate article. Recently, Gu and Van Deun (2018) studied a few model selection methods for regularized SCA and found that a relatively lesser known, computationally efficient method, namely the Index of Sparseness (Gajjar et al., 2017; Trendafilov, 2014; Zou et al., 2006), outperformed cross-validation in terms of selecting the proper component loading structure. Thus, a comprehensive, yet computationally feasible model selection procedure for deciding *R*, *λ*_*L*_, and *λ*_*G*_ based on the Index of Sparseness may be promising, but in this article we refrain from discussing it, because the procedure requires development and validation via, for example, simulation studies.

## The RegularizedSCA package

In this section, we use a small dataset, referred to as the “Herring” data, included in the package because of its didactic value. We present an empirical example in the next section. The following code loads the package and its accompanying dataset “Herring”. The data are originally from Bro et al., (2002) and Nielsen et al., (1999).

R> library(RegularizedSCA) R> names(Herring)

The “Herring” data consist of two small datasets. The “Herring_ChemPhy” dataset contains physical and chemical changes of 21 salted herring samples in a ripening experiment. The “Herring_Sensory” dataset contains the same 21 samples’ sensory data (such as the smell and the sweetness of the herring). Researchers in chemometrics and food sciences are interested in whether certain physical or chemical changes in herring (such as protein level) are associated with certain sensory characteristics (such as sweetness). Thus, we perform a joint analysis on these two datasets and inspect the association between the two datasets by means of their common and distinctive components.

The first step is to pre-process the data by using the function pre_process (that is, to standardize each column over the rows) and then to concatenate the data.

R> ChemPhy <– pre_process(Herring$Herring_ChemPhy) R> Sensory <– pre_process(Herring$Herring_Sensory) R> herring_data − num_var <– cbind(dim(ChemPhy) [2], dim(Sensory)[2])

pre_process can automatically handle missing data by using multiple imputation. In addition, when the number of variables in one block is much larger than another block, it is likely that the information in the former block dominates the latter block. We recommend weighting each block by taking into account the number of variables (e.g., Van Deun et al., 2009, for details). This is done by using the argument weight in pre_process (e.g., pre_process(DATA, weight = TRUE)). In the last line of the code above, we record the number of variables (i.e., columns) per data block, which is used later. We conduct the joint analysis by using models 2, 4, and 5 (see Fig. 2) to illustrate all the important functions in RegularizedSCA. We emphasize that in practice it is not necessary to apply multiple models; users typically choose only one model, and the choice is based on common practice in their research fields and existing literature.

### Joint analysis using model 2

Model 2 states that the number of components *R* is decided by means of the VAF method, the component structure is identified by means of the DISCO-SCA method, and there is some sparseness within the common and distinctive components, determined by tuning *λ*_*L*_. To use the VAF method, we evaluate the following function:

R> vaf <– VAF(DATA = herring_data, Jk = num_var, R = 10) R> summary(vaf)

We have let the function evaluate the proportion of VAF, if there would be *R* = 10 components in the concatenated data. The VAF function displays the proportion of VAF per block and per component in each block. We primarily focus on the component part. In the first block (i.e., the “Herring_ChemPhy” data), the first three components explain most of the information of the block (42.2, 31.6, and 12.8%, respectively), whereas the remaining components explain much less information. In the second block (i.e., the “Herring_Sensory” data), the first four components explain most of the information (55.1, .9, .9, and 12%, respectively). Thus, taking two blocks together, we may conclude that at most four components are needed for further analysis. As a side note, despite that the fourth component in the first block accounts for a much smaller variance than the first three components, it has to be retained for further analysis because we decide to retain four components for the second block.

Next, we use the DISCO-SCA method to identify the component structure, given *R* = 4:

R> discoresult <– DISCOsca(DATA = herring_data, R = 4, Jk = num_var) R> summary(discoresult)

**Fig. 3:**
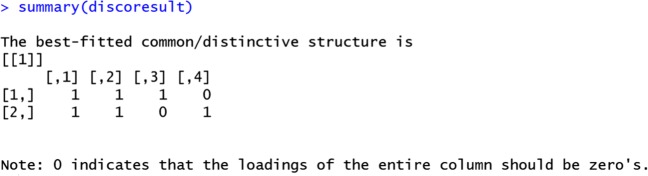
A screenshot of the result of the DISCO-SCA method

Figure 3 presents the screenshot of the result of the DISCO-SCA method. By evaluating summary(discoresult), the program produces a matrix of 1’s and 0’s indicating (non-sparse) common and distinctive components. The matrix has two rows, with the first row representing the first data block (i.e., the “Herring_ChemPhy” data) and the second row representing the second data block (i.e., the “Herring_Sensory” data). The four columns represent the four components (i.e., *R* = 4). The element in the first row and the first column of the matrix is a “1”, meaning that all the component loadings in the first component in the first block may be non-zero loadings. The element in the first row and the fourth column is a “0”, meaning that all the loadings in the fourth component in the first block may be zero loadings. The remaining elements in the matrix are interpreted in the same way. Thus, the matrix suggests that there are two common components (i.e., the first two columns) and two distinctive components (i.e., the remaining two columns).

We now use the regularized SCA model with known component structure (generated by DISCO-SCA) and *λ*_*L*_, which in RegularizedSCA is realized by the functions structuredSCA and cv_structuredSCA. Note that the former function requires the user to specify a value for *λ*_*L*_, whereas the latter function uses *K*-fold (by default, 10-fold) cross-validation to decide the proper value for *λ*_*L*_. Here we use cv_structuredSCA first. cv_structuredSCA (and also structuredSCA) requires the user to specify the component structure, which in this case is generated by the DISCO-SCA method (also see Fig. 3):

“Herring_ChemPhy”: 1 1 1 0 “Herring_Sensory”: 1 1 0 1.

Thus, in R, we specify the component structure, which we refer to as a *target matrix*, as follows:

R> targetmatrix <– matrix(c(1, 1, 1, 1, 1, 0, 0, 1), nrow = 2, ncol = 4)

which is simply the matrix in Fig. 3. Next, we perform a joint analysis with 10-fold cross-validation.

R> maxLasso <– maxLGlasso(DATA = herring_data, num_var, R = 4)$Lasso R> set.seed(115) R> results_cvS <– cv_structuredSCA(DATA = herring_data, Jk = num_var, R = 4, Target = targetmatrix, Position = c(1, 2, 3, 4), LassoSequence = seq(from = 0.0000001, to = maxLasso, length.out = 200)) R> plot(results_cvS)

Note that in the code above, we use the function maxLGlasso to decide the smallest value for *λ*_*L*_, denoted by $\lambda ^{max}_{L}$, that makes the entire concatenated component loading matrix a zero matrix (i.e., **P**_*C*_ ≡**0**). Thus, sparse results are found when *λ*_*L*_ is between 0 and $\lambda ^{max}_{L}$. The algorithm goes through a sequence of 200 evenly spaced values from 0 to $\lambda ^{max}_{L}$ and performs 10-fold cross-validation. Three comments are in order regarding the cv_structuredSCA function. First, if the LassoSequence argument is missing, the algorithm will first run maxLGlasso internally and then perform cross-validation on a sequence of 50 (instead of 200) even spaced values from 0 to $\lambda ^{max}_{L}$. The Position argument specifies which component(s) is estimated with the Lasso penalty. Here, Position = c(1, 2, 3, 4) means that the Lasso penalty is imposed on all four components. If, for example, the user defines Position = c(1, 3), then the first and third components will be estimated with the Lasso penalty, resulting in a sparse common component (i.e., the first component), a non-sparse common component (i.e., the second component), a sparse distinctive component (i.e., the third component), and a non-sparse distinctive component (i.e., the fourth component). Third, by default the algorithm performs a 10-fold cross-validation, but another number of folds can be specified (see the help documentation in RegularizedSCA).

**Fig. 4:**
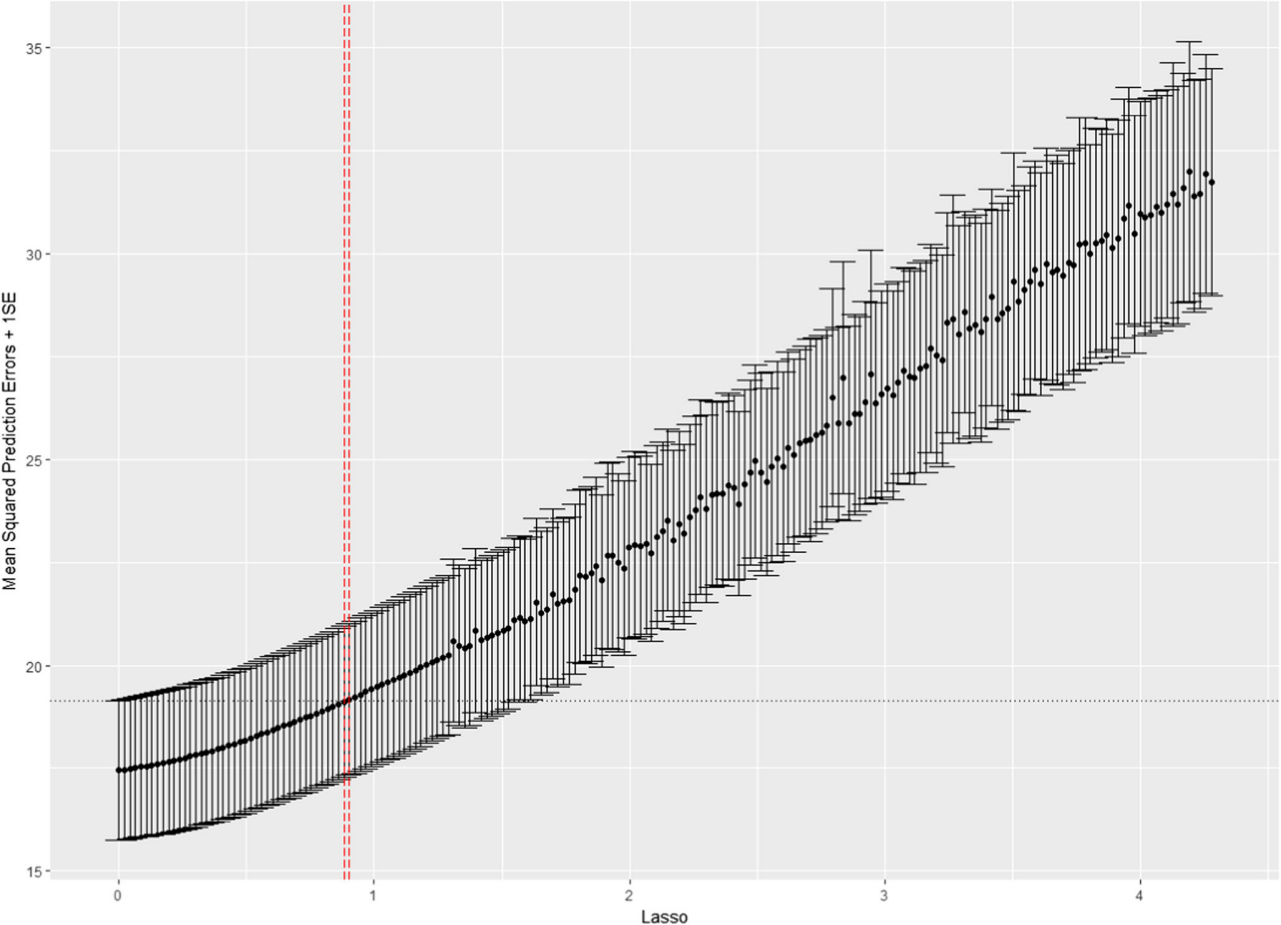
The cross-validation curve

Figure 4 displays the cross-validation curve, generated by plot(results_cvS). The region between the vertical red dashed lines in the figure indicates the region for proper Lasso tuning parameters based on the “one-standard-error” rule (which is indicated by the vertical black dotted line), and the region of proper Lasso tuning parameters can be obtained as follows:

R> results_cvS$LassoRegion,

The region is between .8814759 and .9029753, and thus a proper Lasso value could be .8922256, the average of the two. We remind readers that the optimal value lies within this region, meaning that if one choose .9029753, the largest of the two, then the estimated component loading matrix may be sparser than the matrix by using the optimal value. Because, in this example, the algorithm uses a sequence of 200 evenly spaced values, the region generated by the algorithm is very small (.9029753-.8814759 = .0214994). Thus, using either the average value (i.e., .8922256) or the larger value (i.e., .9029753) does not drastically influence the final result. Using the smaller value (i.e., .8814759) may be a safer choice. Alternatively, users may also ask for a Lasso value that is recommended by the algorithm by calling for summary(results_cvS), but we remind readers that the recommended value is the one whose MSPE is closest to (i.e., could be slightly larger or smaller than) the smallest MSPE plus one standard error. Users who prefer a value whose MSPE is closest to and smaller than the smallest MSPE plus one standard error may consult the full report by using summary(results_cvS, disp = “full”). We now re-run the analysis with *λ*_*L*_ = .8922256:

R> set.seed(115) R> result_str <– structuredSCA(DATA = herring_data, Jk = num_var, R = 4, Target = targetmatrix, Position = c(1, 2, 3, 4), LASSO = 0.8922256)

The Lasso not only puts component loadings exactly to zero but also shrinks each of the non-zero loadings towards zero. Such shrinkage of the non-zero loadings can be undone as follows:

R> final_comLoadingS <– undoShrinkage(DATA = herring_data, R = 4, Phat = result_str$Pmatrix) R> summary(final_comLoadingS)

**Fig. 5:**
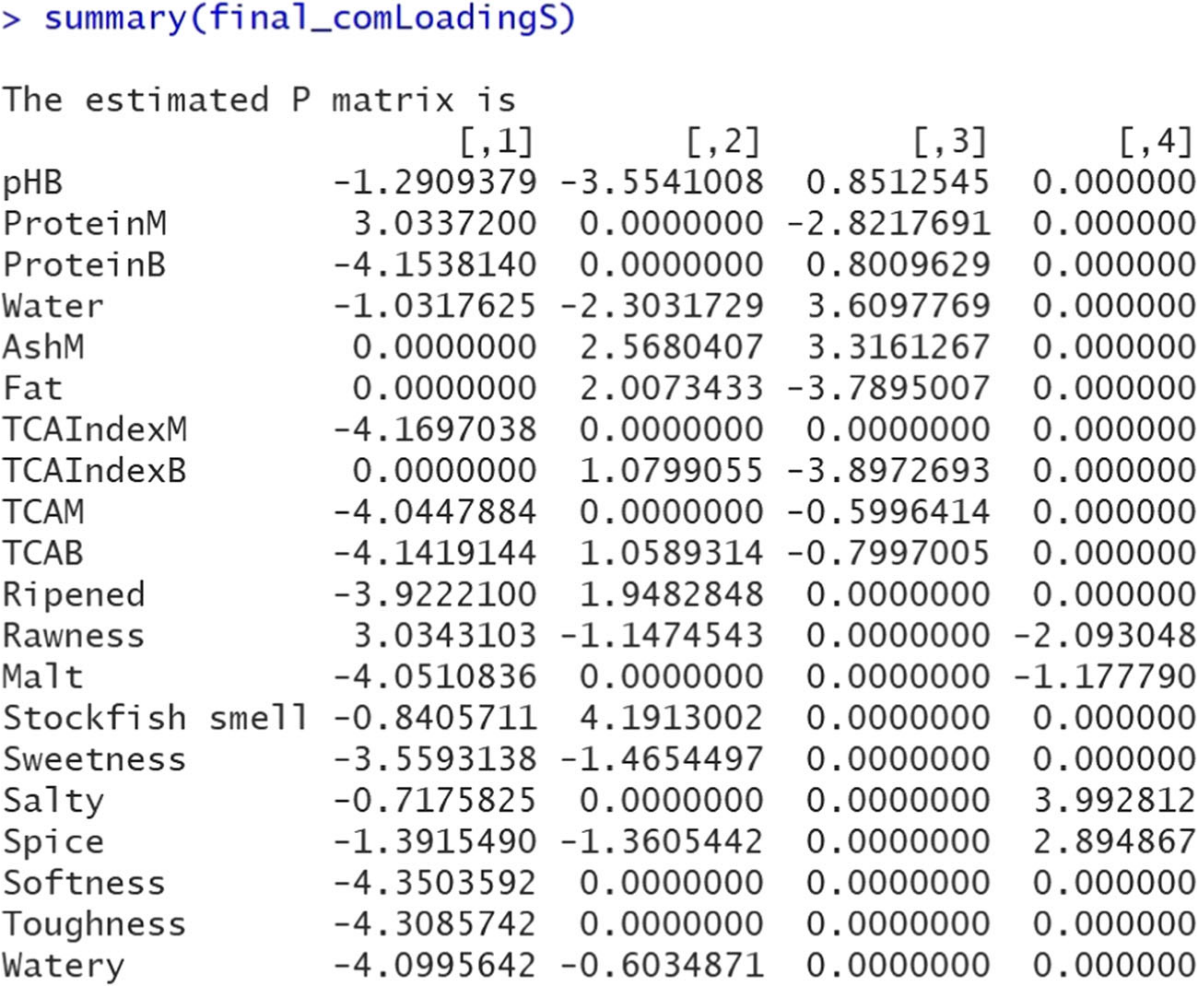
A screenshot of the output of the estimated loadings (Model 2). Note that the final re-estimated non-shrinkage component loading matrix $\hat {\mathbf {P}}$ automatically includes row names if the raw data contains variable names

**Fig. 6:**
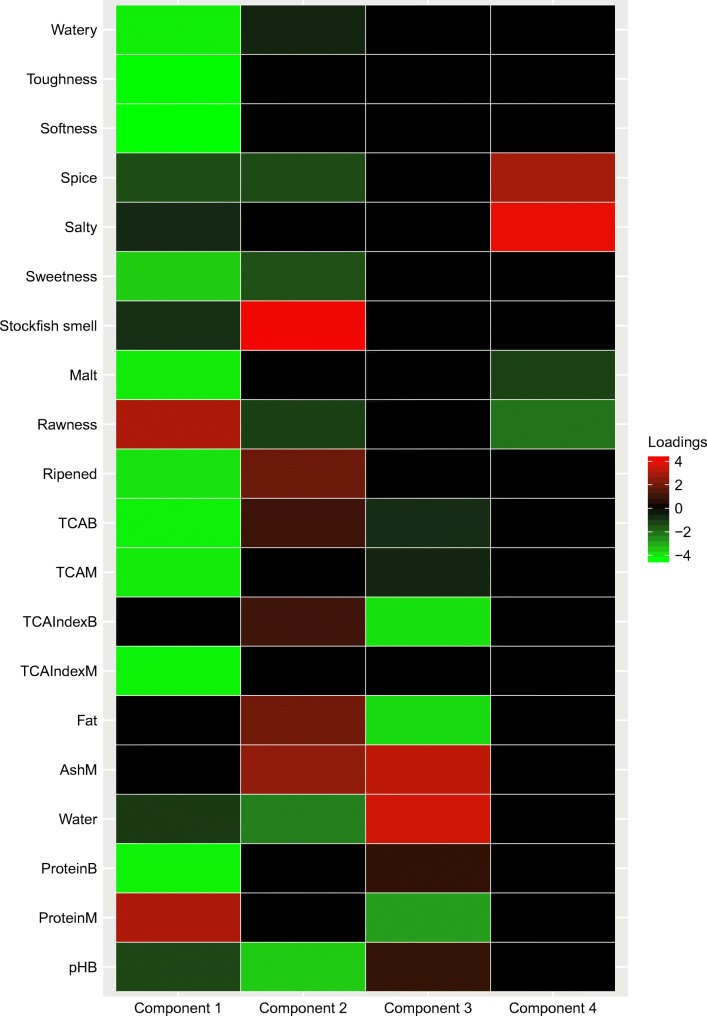
A heatmap of the component loading matrix (Model 2). The first ten rows represent the loadings from the “Herring_Sensory” data (hence the second block), and the remaining ten rows represent the loadings from the “Herring_ChemPhy” data (hence the first block)

Now we obtain a component loading matrix with combination of common and distinctive components as defined previously, and meanwhile there is some sparseness within the common and distinctive components. Figure 5 presents the screenshot of the output of summary(final_comLoadingS). In some research fields, such as chemometrics and genomics, a heatmap of the component loading matrix $\hat {\mathbf {P}}$ is often used to interpret the loadings (see Fig. 6). Figure 6 shows that the first two components are sparse common components, where both data blocks contribute information, whereas the last two components represent the sparse distinctive processes that are not shared across blocks. As a side note, the heatmap function is not included in the RegularizedSCA package, since other packages, such as ggplot2 (Wickham, 2009), have already provided adequate functions for plotting heatmaps.

### Joint analysis using model 4

Model 4 states that the number of components *R* is decided by means of the VAF method (*R* = 4 for the “Herring” data), the component structure is identified by *λ*_*G*_, and the regularized SCA model with *λ*_*L*_ and *λ*_*G*_ is used. The 10-fold cross-validation for *λ*_*L*_ and *λ*_*G*_ is performed by evaluating the following code:

R> set.seed(115) R> results_cv <– cv_sparseSCA(DATA = herring_data, Jk = num_var, R = 4)

Note that if not specified otherwise, cv_sparseSCA performs 10-fold cross-validation, with a sequence of 20 Lasso tuning parameters and 20 Group Lasso tuning parameters (from .00000001 to the smallest tuning parameter value that makes all the component loadings equal to zero). Users may also use maxLGlasso and specify a sequence of Lasso and Group Lasso tuning parameters by themselves. In addition, the Group Lasso penalty is applied to each component separately, which is the component-wise method mentioned in the Method section. As an aside, the user may also impose the Group Lasso penalty on an entire data block (i.e., the block-wise method) by calling cv_sparseSCA(DATA = herring_data, Jk = num_var, R = 4, method = “datablock”). By evaluating the following command

R> summary(results_cv)

we obtain the recommended Lasso tuning parameter (*λ*_*L*_ = 1.503148) and Group Lasso tuning parameter (*λ*_*G*_ = .3655355) based on the “one-standard-error” rule. Users may also consult summary(results_cv, disp = “full”) to get a full view of results of the cross-validation procedure, which includes, for example, the values of tuning parameters that have been evaluated, and mean squared prediction errors etc. We remind readers that the recommended Lasso and Group Lasso values here are the ones whose MSPE is closest to (i.e., could be slightly larger or smaller than) the smallest MSPE plus one standard error. Users may also consult summary(results_cv, disp = “full”) to identify a pair of Lasso and Group Lasso values whose MSPE is closest to but smaller than the smallest MSPE plus one standard error. We run the final model with the recommended tuning parameters *λ*_*L*_ = 1.503148, *λ*_*G*_ = .3655355, and *R* = 4, and check the estimated component loading matrix $\hat {\mathbf {P}}$ for its sparseness.

R> set.seed(115) R> final_results <– sparseSCA(herring_data, num_var, R = 4, LASSO = 1.503148, GROUPLASSO = 0.3655355, NRSTART = 20)

Because both the Lasso and the Group Lasso shrink the non-zero component loadings towards zeros, the shrinkage may be undone as follows:

R> final_Loading <- undoShrinkage(herring_data, R = 4, Phat = final_results$Pmatrix) R> summary(final_Loading)

**Fig. 7:**
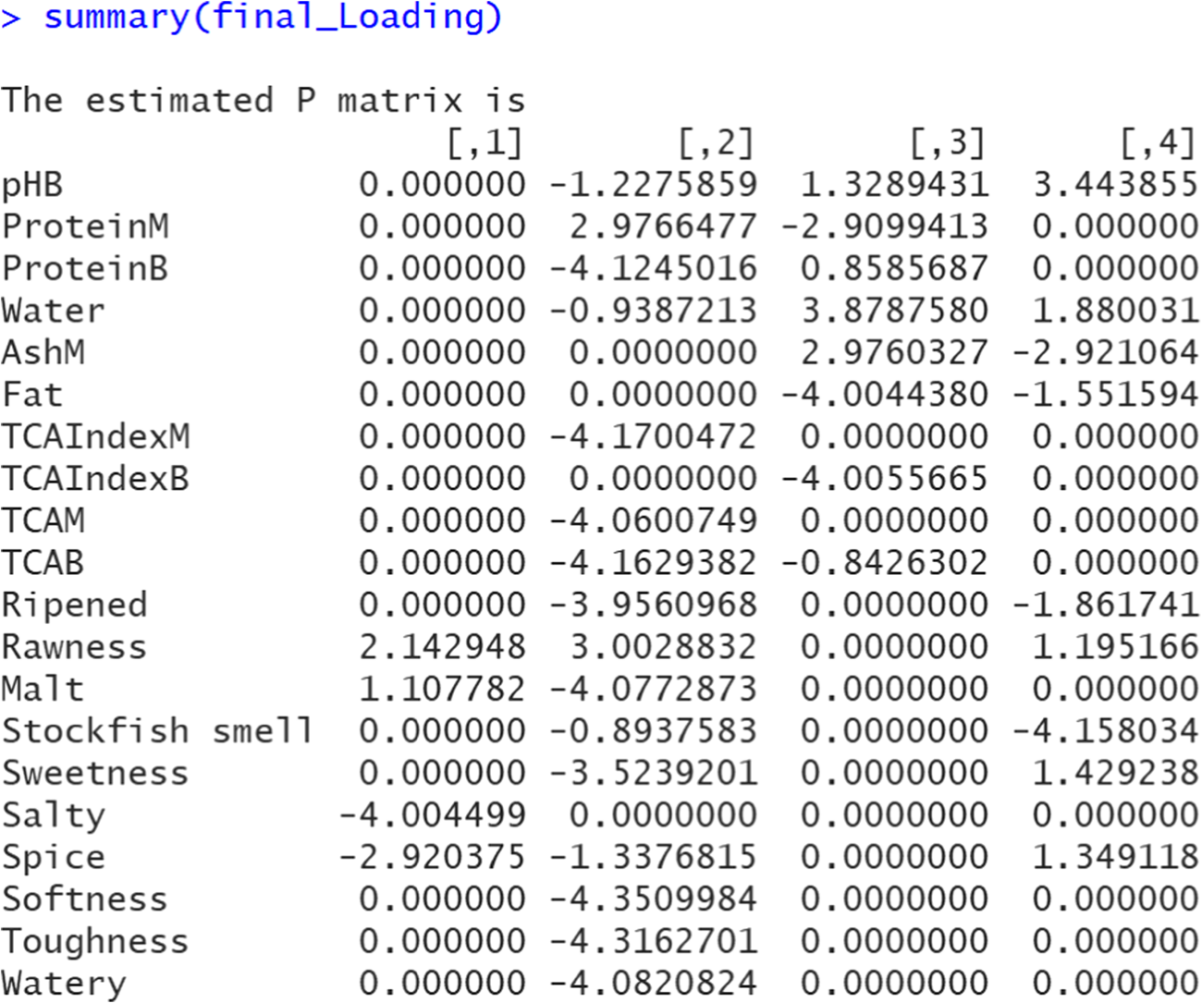
A screenshot of the output of the estimated loadings (Model 4). Note that the final re-estimated non-shrinkage component loading matrix $\hat {\mathbf {P}}$ automatically includes row names if the raw data contains variable names

**Fig. 8:**
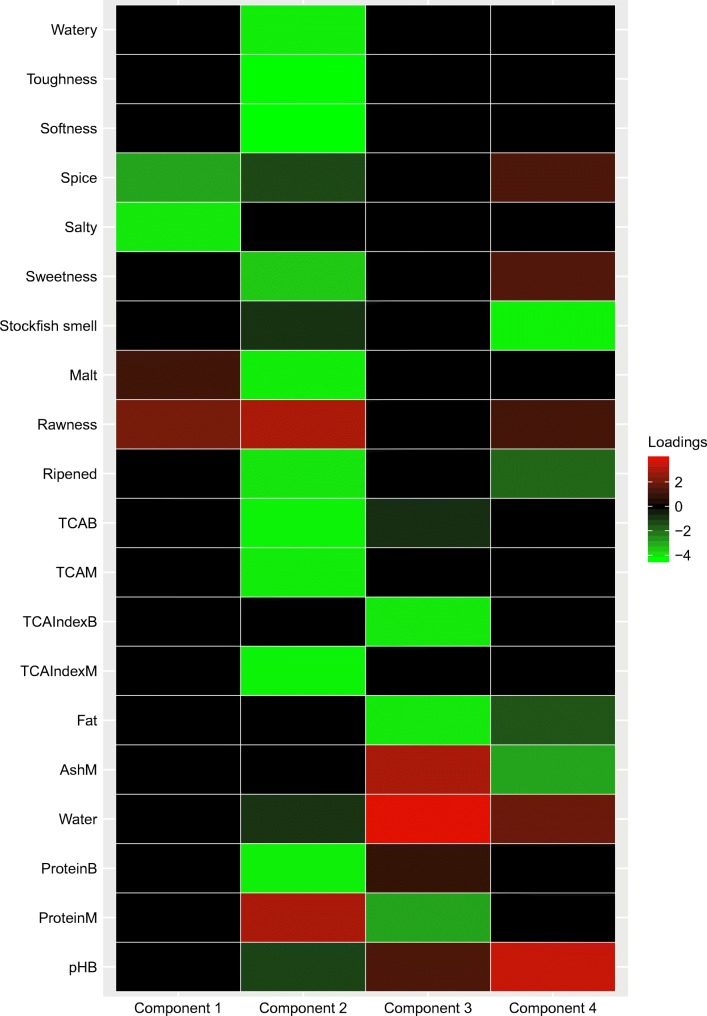
A heatmap of the estimated component loading matrix (Model 4). The first ten rows represent the loadings from the “Herring_Sensory” data (hence the second block), and the remaining ten rows represent the loadings from the “Herring_ChemPhy” data (hence the first block)

Figure 7 presents the screenshot of the output of summary(final_Loading). We also include the heat map (see, Fig. 8). Comparing Figs. 6 and 8, one may notice that the results are very close. The components switch positions and signs due to invariance of the regularized SCA solution under permutations and reflections of the components. However, the results cannot be identical, because two different models are used after all. Readers may notice that the optimal *λ*_*L*_ for Model 2 is approximately .89, and that for Model 4 is 1.50. We do not expect that the *λ*_*L*_s for the two models are of similar magnitude, because in Model 4, both the Lasso and the Group Lasso cause shrinkage, but in Model 2 the Lasso has to account for all the shrinkage.

One may notice that, for Model 4, a cross-validation curve like Fig. 4 is not available, because the cross-validation procedure involves a grid of *λ*_*L*_ and *λ*_*G*_. A possible solution is to provide a series of cross-validation curves conditional on *λ*_*G*_s, and therefore for each *λ*_*G*_ a cross-validation curve is presented. But this solution is problematic for two reasons. First, imagine a sequence of 100 *λ*_*G*_s is evaluated by the algorithm, then 100 cross-validation curves have to be provided, making interpretation difficult. Second, based on user feedback, we noticed that users were often confused by this conditional approach.

### Joint analysis using model 5

Model 5 states that the number of common and distinctive components and correspondingly the component structure are identified by means of the PCA-GCA method. Afterwards, the regularized SCA model with known component structure and *λ*_*L*_ ≡ 0 is used to estimate the component loadings and scores. Because *λ*_*L*_ ≡ 0, there is no sparseness within the common/distinctive components.

To use the PCA-GCA method, we first evaluate the following function:

16 $$ \textsf{R\(>\) pca\_gca(DATA \(=\) herring\_data, Jk \(=\) num\_var)} $$

**Fig. 9:**
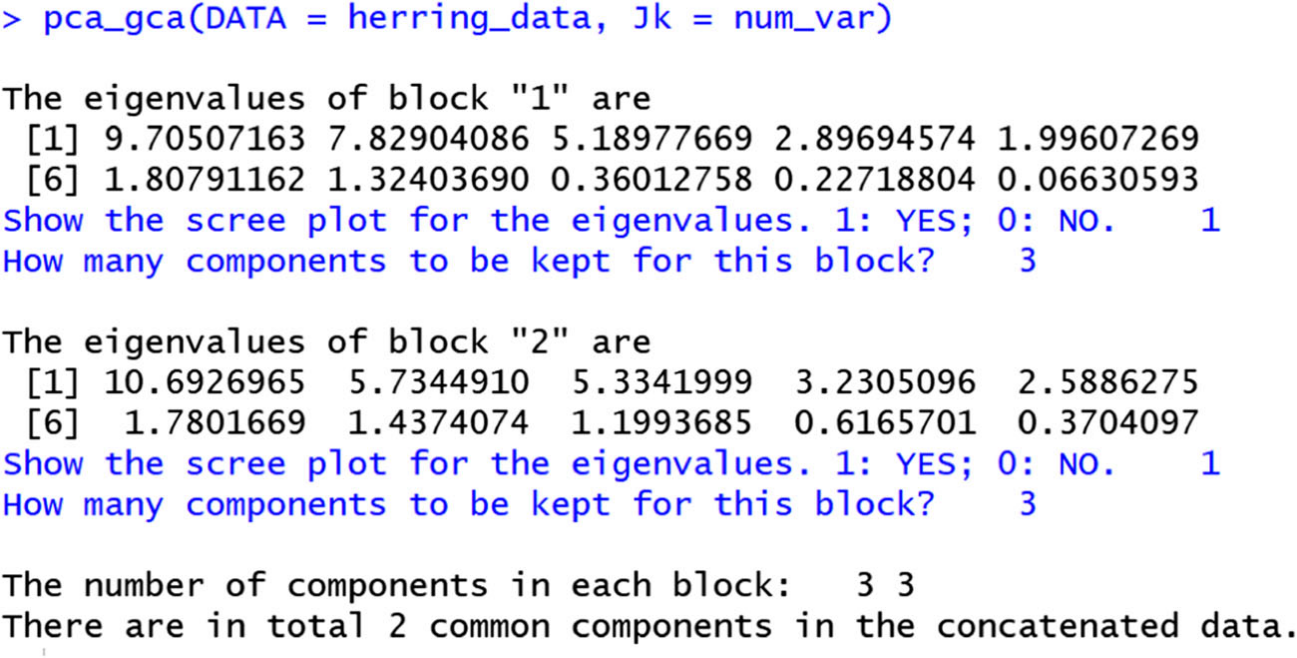
The user–computer interaction procedure for the PCA-GCA method

The pca_gca function incorporates a user–computer interaction procedure (see the screenshot in Fig. 9): The function first performs PCA on each data block, and then presents the eigenvalues and also a scree plot to the user. The user must tell the program whether she would like to see the scree plot. (We advise the user to see the scree plot.) Afterwards, the user tells the program how many components should be retained for each block based on the eigenvalues and the scree plots. The next step in the PCA-GCA procedure, automated by the package, is to decide the number of common and distinctive components. Here, the default is to consider a component to be common if the correlation between two components, one from the “Herring_ChemPhy” block and the other from the “Herring_Sensory” block, is higher than .7. The user may change the default value. We emphasize that, more research is needed on the correlation threshold. Here we followed Smilde et al. (2017, p. 15) and used .7 as the threshold.

**Fig. 10:**
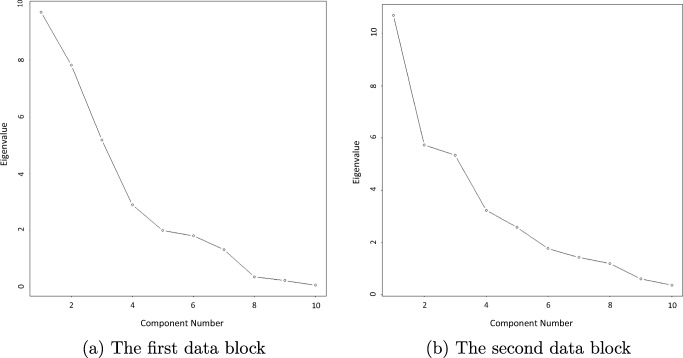
The scree plots generated by the pca_gca function

The output (see, Fig. 9) starts with presenting the eigenvalues of the first block, and then the program asks whether to show the scree plot. We answer “yes” by entering “1” on the keyboard, and then a scree plot is shown (see, Fig. 10). Afterwards, the program asks how many components to retain, and we enter “3”, based on the eigenvalues and the scree plot. The program then moves on to the second block and repeats the aforementioned procedure. In the end, pca_gca tells us that in each block there are three components, but there are two common components shared by the two blocks (see, Fig. 9). This means that, for each block, there are two common components and one distinctive component, and thus there should be *R* = 4 components in total in the integrated data.

Thus, a possible component structure (i.e., target matrix) could be

“Herring_ChemPhy”: 1 1 1 0 “Herring_Sensory” : 1 1 0 1,

whose corresponding target matrix is matrix(c(1, 1, 1, 1, 1, 0, 0, 1), nrow = 2, ncol = 4). We emphasize that, because of invariance of the DISCO-SCA solution under permutations of components, the component structure proposed above is one of the many equivalent structures. Another possible component structure, by switching the position of the first and third columns, is

“Herring_ChemPhy”: 1 1 1 0 “Herring_Sensory” : 0 1 1 1,

whose corresponding target matrix is matrix(c(1, 0, 1, 1, 1, 1, 0, 1), nrow = 2, ncol = 4). Because the structures are equivalent, the resulting estimated component scores and loadings are identical, after switching the columns and/or signs of loadings. In other words, both target matrices above can be used. We now use the first target matrix and estimate the final model.

R> targetmatrix <– matrix(c(1, 1, 1, 1, 1, 0, 0, 1), nrow = 2, ncol = 4) R> set.seed(115) R> result_strModel5 <– structuredSCA(DATA = herring_data, Jk = num_var, R = 4, Target = targetmatrix, LASSO = 0) R> final_LoadingModel5 <– undoShrinkage(herring_data, R = 4, Phat = result_strModel5$Pmatrix) R> summary(final_LoadingModel5)

**Fig. 11:**
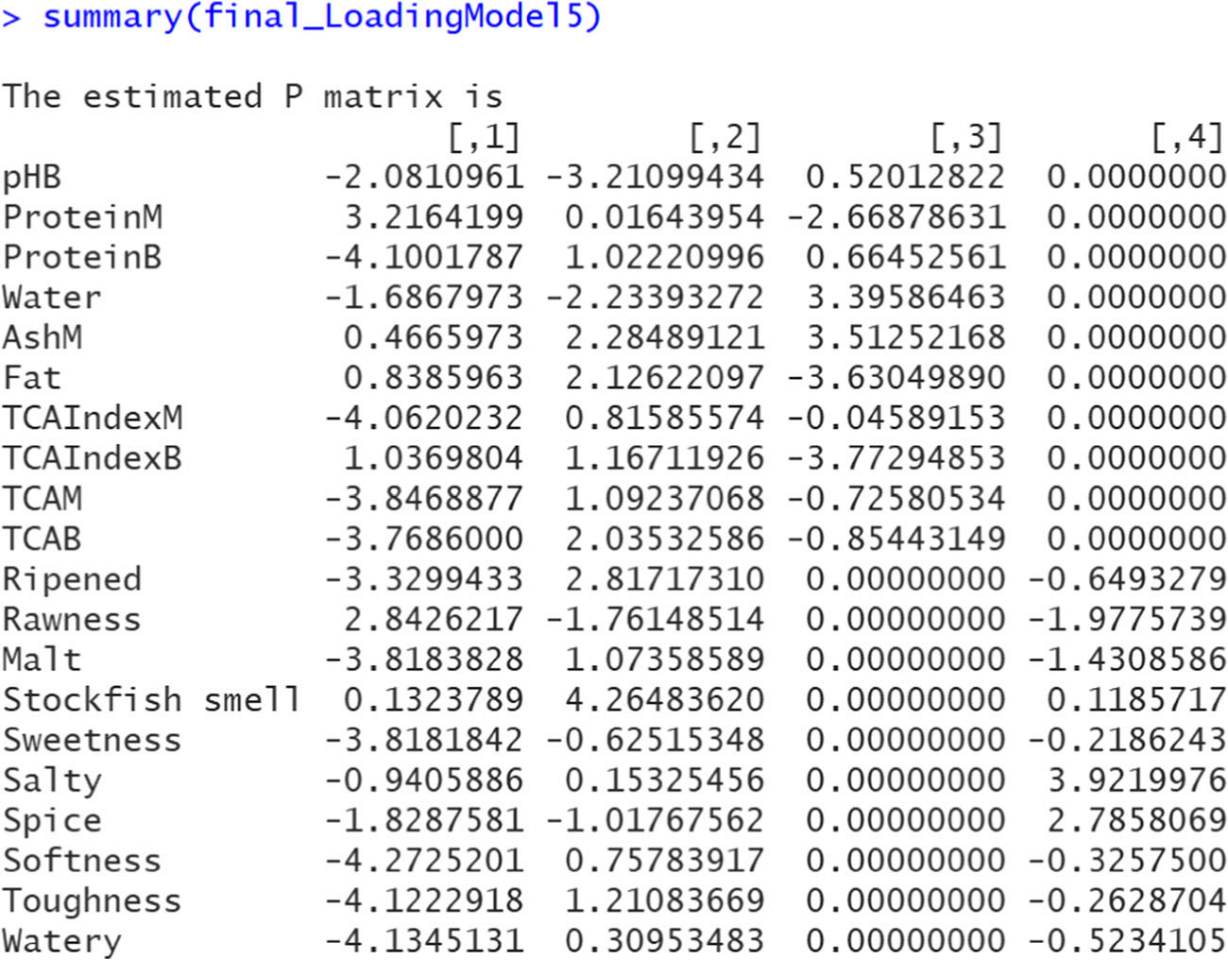
A screenshot of the output of the estimated loadings (Model 5). Note that the final re-estimated non-shrinkage component loading matrix $\hat {\mathbf {P}}$ automatically includes row names if the raw data contains variable names

A screenshot of the estimated loadings can be found in Fig. 11. There is no sparseness within the common and distinctive components, as desired. The heatmap is omitted.

Before concluding this section, we mention two points about the specification of the target matrix. First, when the PCA-GCA method is used, because one chooses the number of components for each block based on PCA, it is normal to have a different number of components per block. For example, imagine that one block with many variables requires ten components, and another block with, say, two variables that requires one component. We further assume that there is no common component, and therefore *R* = 10 + 1 = 11. In this case, one may specify the target matrix as follows:

Block 1: 1 1 1 1 1 1 1 1 1 1 0 Block 2 : 0 0 0 0 0 0 0 0 0 0 1,

provided that *R* is smaller than the number of subjects. Second, the specification of the target matrix can be easily done by calling the (summary of) DISCOsca function as shown before. When more than two blocks are to be analyzed jointly, we recommend using DISCOsca, because this function can directly analyze more than two data blocks.

## An empirical application

To illustrate the usefulness of RegularizedSCA, we present an analysis of empirical data on parent–child relationship, which readers in behavioral sciences are familiar with. We use this example to show how information regarding parent–child relationship can be obtained by examining the components estimated by the model.

In psychological, sociological, educational, and medical research, researchers are often interested in the relation between parents’ behavior and children’s behavior (e.g., Frome & Eccles, 1998; Moore et al., 1991; Sharpley, Bitsika, & Efremidis, 1997; Cummings & Davies, 1995; Acock, 1984; Trost et al., 2003). In this section, we conduct a regularized SCA analysis on survey data of 195 families, which were originally from the dataset entitled “The 500 Family Study” (Schneider and Waite, 2008). Three hundred and five families were removed because they contained many missing entries. For each family, the parents filled in eight questionnaires, and their child filled in seven questionnaires (see, Table 1), regarding their feelings, recent activities, and their opinions about relationship, etc. For each questionnaire, a sum score is computed. Thus, the mother, father, and child datasets contain 8, 8, and 7 scores, respectively. The supplementary material contains the R script for running the analysis. For obtaining the data from the original “500 Family Study”, please see https://github.com/ZhengguoGu/paperRegularizedSCA/blob/master/ForAuthorsOnly/500FamilyData.Rmd.

**Table 1:** Descriptive statistics of the 195 family data

Questionnaire title	Mean	SD
Mother		
Relationship with partners (the higher the score, the more satisfied)	3.58	.79
Argue with partners (the higher the score, the less violent)	3.65	.42
Child’s bright future (the higher the score, the stronger the feeling of bright future)	4.49	.52
Activities with the child (the higher the score, the more activities)	2.40	.39
Feelings about parenting (the higher the score, the more positive about parenting)	3.33	.68
Communication with the child (the higher the score, the more communication)	4.16	.50
Argue (aggressively) with the child (the higher the score, the less aggressive)	3.08	.45
Confidence about oneself (the higher the score, the more confident)	2.71	.43
Father		
Relationship with partners (the higher the score, the more satisfied)	3.67	.70
Argue with partners (the higher the score, the less violent)	3.77	.42
Child’s bright future (the higher the score, the stronger the feeling of bright future)	4.48	.51
Activities with the child (the higher the score, the more activities)	2.30	.38
Feelings about parenting (the higher the score, the more positive about parenting)	3.40	.64
Communication with the child (the higher the score, the more communication)	3.97	.60
Argue (aggressively) with the child (the higher the score, the less aggressive)	3.18	.42
Confidence about oneself (the higher the score, the more confident)	2.78	.47
Child		
Self confidence/esteem (the higher the score, the more confident)	2.08	.46
Academic performance (the higher the score, the better the performance)	6.87	1.32
Social life and extracurricular activities (the higher the score, the more social life)	2.22	.38
Importance of friendship (the higher the score, the more important friendship is)	3.94	.61
Self image (the higher the score, the more positive self image is)	2.56	.52
Happiness (the higher the score, the happier)	2.29	.44
Confidence about the future (the higher the score, the more confident about the future)	3.94	.47

We performed the regularized SCA analysis on the concatenated data matrix consisting of three data blocks for the mothers, the fathers, and the children, respectively; thus, the data matrices were concatenated with respect to the same family unit. To choose the appropriate model, we resorted to Fig. 2. In the first step, we decided to use the VAF method, because this method could be readily applied to the three data blocks. We decided to retain five components. In the second step, we used cross-validation to identify the component structure. Note that for this step, one may also use the DISCO-SCA method. In the last step, we also used cross-validation to achieve some sparseness within the common/distinctive components. Therefore, we used Model 4 in Fig. 2. The estimated component loading matrix is presented in Table 2: The first component is a sparse common component. The second and third components are sparse distinctive component specific for parents. The fourth component is a distinctive component specific for children. The last component is a sparse distinctive component specific for fathers. The first, second, and third components are of particular interest because they reveal the relation between parents and children (the first component) and between parents themselves (the second and third components). To interpret the table, we take the first and the third components for illustration. The first component shows that a child’s higher self-confidence is positively associated with parents’ higher confidence in the child’s future, parents’ more positive feeling about parenting, parents’ less aggressiveness during arguments with the child, mother’s being less violent during arguments with the father, mother’s more communication and activities with the child, and mother’s higher self-confidence. The third component suggests that more satisfaction in the relationship and less aggressive behavior during an argument with the partner go together with more confident feelings about oneself; this relationship holds for both mothers and fathers.

**Table 2:** The estimated component loading matrix of the 195 family data

	Component 1	Component 2	Component 3	Component 4	Component 5
Mother					
Relationship with partners	0	0	11.92	0	0
Argue with partners	− 5.53	0	5.88	0	0
Child’s bright future	− 8.83	0	0	0	0
Activities with children	− 4.65	− 9.02	0	0	0
Feeling about parenting	− 9.02	0	0	0	0
Communication with children	− 9.20	0	0	0	0
Argue with children	− 8.78	0	0	0	0
Confidence about oneself	− 6.66	0	7.26	0	0
Father					
Relationship with partners	0	0	11.80	0	0
Argue with partners	0	0	5.26	0	− 9.17
Child’s bright future	− 3.39	0	0	0	− 5.76
Activities with children	0	− 11.56	0	0	0
Feeling about parenting	− 4.04	0	0	0	− 6.94
Communication with children	0	− 8.17	0	0	0
Argue with children	− 4.98	0	0	0	− 9.88
Confidence about oneself	0	0	5.60	0	− 8.19
Child					
Self confidence/esteem	− 5.82	0	0	8.66	0
Academic performance	0	0	0	7.08	0
Social life and extracurricular	0	0	0	4.10	0
Importance of friendship	0	0	0	9.60	0
Self Image	0	0	0	10.36	0
Happiness	0	0	0	9.55	0
Confidence about the future	0	0	0	7.48	0

*Note*. To interpret the loadings, we compare the signs of the loadings of each block within a component. Take Component 1 for example, all the non-zero loadings are of the same sign (in this case, ‘-’ sign), meaning that the variables corresponding to those loadings are positively associated with each other; that is, the higher a mother scores on, for example, “Argue with partners”, the higher she scores on the remaining variables (excluding “Relationship with partners”), and also the higher her partner (i.e., the father) scores on “Child’s bright future”, “Feeling about parenting”, and “Argue with children”, and also the higher the child scores on “Self confidence/esteem” and “Self image”

This empirical example shows that the regularized SCA approach to multiblock analysis can provide an interesting insight in dyadic relationship between parents and children and between parents themselves. One may notice that the regularized SCA approach (and SCA in general) does not provide information regarding the directionality of the dyadic relationship, and thus if directionality is of primary interest, readers are advised to use other methods, such as directional network models.

## Concluding remarks

With an increasing trend in using large datasets coming from multiple sources, data integration tools that yield insights in joint and specific sources of variation and select the important variables therein are of crucial importance. The package proposed here fulfills this need. The functions in this package are flexible, and they cover important methods for identifying common and unique information in datasets. RegularizedSCA is available from the Comprehensive R Archive Network (CRAN) at https://cran.r-project.org/web/packages/RegularizedSCA/index.htmlhttps://cran.r-project.org/web/packages/RegularizedSCA/index.html. RegularizedSCA, as far as we know, is the first R package that focuses on data integration from the (regularized) simultaneous component perspective.

The Regularized SCA approach to data integration is a fruitful field for future research. At this moment, little is known about which model selection method(s) are suitable for regularized simultaneous component analysis: Cross-validation is a popular choice, but it is known that cross-validation methods tend to retain more variables than needed (Chen & Chen, 2008). Other model selection methods, such as Index of Sparseness (Gajjar et al., 2017; Trendafilov, 2014; Zou et al., 2006), stability selection (Meinshausen & Bühlmann, 2010), and AIC, BIC type methods (e.g., Chen & Chen, 2008; Croux,Filzmoser, & Fritz, 2013; Guo, James, Levina, Michailidis, & Zhu, 2010), may be considered as alternative methods for model selection. Furthermore, regularized SCA needs to be further extended to incorporate categorical data, which are often seen in social and behavioral research.
